# Under the shade of world events: a never-ending crisis in Yemen

**DOI:** 10.1016/j.eclinm.2023.102302

**Published:** 2023-10-23

**Authors:** eClinicalMedicine

Yemen, a country situated in the southern part of the Arabian Peninsula that was divided into two Yemeni states (North and South) by the British and the Ottoman Empire in the 1800s, underwent a unification process in 1990. The merger involved the Saudi Arabia-backed Yemen Arab Republic (North Yemen) and the Soviet-backed People's Democratic Republic of Yemen (South Yemen). However, due to religious, cultural, and political differences, the ambitious goal of creating a unified nation faced challenges. The Southern Movement’s pursuit of autonomy, the territorial gains by Al-Qaeda in the Arabian Peninsula in the south and east, and the rebellion led by the Houthi movement further destabilised the country. In 2014, the Houthi rebels, a Shiite movement that has been fighting Yemen’s Sunni-majority government since 2004, took control of Yemen's capital, Sana’a, triggering a civil war and exacerbating one of the most significant humanitarian crises of our time.

The conflict in Yemen has had a devastating impact on the population, with over 21 million people in need of humanitarian assistance. Urgent health services are required by 20.3 million individuals, while 17.3 million suffer from acute food insecurity, and 15.3 million have no access to clean water, sanitation, and hygiene. Women and children bear the greatest burden of the conflict’s consequences, particularly in areas controlled by the Houthi group. Women have experienced a reduction in their rights, including freedom of expression, access to health care, and employment while facing increasing restrictions. Scarce work opportunities force women to shoulder the responsibility of caring for their families, and girls are often forced to abandon their education for early marriages. Socioeconomical gender disparities, discriminatory legal systems, and economic inequality have made women and girls vulnerable to violence and abuse.

The crisis has resulted in a massive vulnerability among children, with over 11,000 killed or injured and many recruited for fighting. Yemen has one of the highest rates of malnutrition, accounting for 45% of deaths among children under 5 years old. More than 2 million children suffer from acute malnutrition, which requires immediate treatment to prevent long-term physical and neurological consequences, as well as the risk of chronic diseases such as cystic fibrosis, chronic renal failure, chronic liver diseases, childhood malignancies, congenital heart disease, and neuromuscular diseases in adulthood.

The health-care system in Yemen continues to deteriorate, and the ongoing civil war has had a substantial impact on public services. Hospitals and clinics have been attacked, damaged, and even destroyed. Additionally, insufficient funding, a shortage of medical staff, and a scarcity of essential equipment and furniture are the main causes of the health system’s decline. Unfortunately, Yemen continues to suffer from poor facilities and not having access to adequate health-care services today, which has significantly increased maternal and infant mortality rates.

Yemen remains one of the most drinking water-scarce countries in the world due to the absence of effective infrastructure, and the ongoing conflict has severely depleted resources in the country, limiting access to adequate sanitation and personal hygiene and thus increasing the risk of infectious diseases. Climate change has fuelled outbreaks of measles, diphtheria, dengue fever, cholera, and polio that have further rocked the country. In 2016, the cholera outbreak led to many deaths and remains the largest cholera epidemic in modern times. More than 20 million people in Yemen live in areas at high risk for malaria, and more than 1 million new cases occur each year. The COVID-19 pandemic has thrown Yemen's health-care system into further turmoil. The subsequent waves of COVID-19 have exacerbated the humanitarian crisis and put even more strain on the already struggling health-care system with the increase in patients who needed new vaccines and special care.

Global organisations are making major efforts to address the human impact of the crisis in Yemen. The UN Office for the Coordination of Humanitarian Affairs has established a Yemen 2023 Humanitarian Response Plan to meet the needs of and provide life-saving emergency assistance to 14 million people. UNICEF supports children through its programmes on the following issues: (1) malnutrition, through the provision of therapeutic staple food and medical supplies; (2) public health system, by integrating mobile teams, community health workers and community midwives, and providing treatments in the area with no functioning health centres; (3) hygiene, by improving water supply and sanitation; (4) child protection, by preventing physical and psychological harm from abuse and violence; and (5) education, by implementing safe school protocols. In 2021, UNICEF has treated over 315,000 children under the age of 5 years for acute malnutrition and has provided primary health care to over 2.9 million children and women.

Despite the humanitarian assistance and a truce agreed upon by the parties under the UN the Yemen crisis continues to persist, with no immediate resolution in sight. The conflict has caused immense suffering and trauma, devastating the lives of millions of Yemeni people. It is crucial for the global governments, the UN, and all parties involved to prioritise the pursuit of peace and actively engage in diplomatic efforts to find a lasting solution. The governments and the UN must play a pivotal role in pushing for peace by engaging in dialogue, and negotiations, and fostering a spirit of compromise. Additionally, safeguarding the rights and wellbeing of children should be of paramount concern throughout the resolution process.

